# The Molecular Mechanisms of Resistance to IDH Inhibitors in Acute Myeloid Leukemia

**DOI:** 10.3389/fonc.2022.931462

**Published:** 2022-06-23

**Authors:** Xiaomei Zhuang, Han Zhong Pei, Tianwen Li, Junbin Huang, Yao Guo, Yuming Zhao, Ming Yang, Dengyang Zhang, Zhiguang Chang, Qi Zhang, Liuting Yu, Chunxiao He, Liqing Zhang, Yihang Pan, Chun Chen, Yun Chen

**Affiliations:** ^1^ Edmond H. Fischer Translational Medical Research Laboratory, Scientific Research Center, The Seventh Affiliated Hospital, Sun Yat-sen University, Shenzhen, China; ^2^ Department of Pediatrics, The Seventh Affiliated Hospital, Sun Yat-sen University, Shenzhen, China

**Keywords:** cancer metabolism, IDH1, IDH2, acute myeloid leukemia, drug resistance

## Abstract

Gain-of-function mutations of isocitrate dehydrogenases 1/2 (IDH1/2) play crucial roles in the development and progression of acute myeloid leukemia (AML), which provide promising therapeutic targets. Two small molecular inhibitors, ivosidenib and enasidenib have been approved for the treatment of IDH1- and IDH2-mutant AML, respectively. Although these inhibitors benefit patients with AML clinically, drug resistance still occurs and have become a major problem for targeted therapies of IDH-mutant AML. A number of up-to-date studies have demonstrated molecular mechanisms of resistance, providing rationales of novel therapeutic strategies targeting mutant IDH1/2. In this review, we discuss mechanisms of resistance to ivosidenib and enasidenib in patients with AML.

## Introduction

Isocitrate dehydrogenases 1/2 (IDH1/2) are metabolic enzymes catalyzing the oxidative decarboxylation of isocitrate to α-KG and reducing NAD(P)+ to NAD(P)H in the tricarboxylic acid (TCA) cycle (1). In human cells, IDH1 localizes to peroxisome and cytoplasm (2, 3) and IDH2 localizes to mitochondria (4). IDH1 plays a prominent role in glucose sensing (5) and lipid metabolism (6), while IDH2 is involved in regulating oxidative respiration. Thus, both IDH1 and IDH2 are thought to play key roles in cellular metabolisms. Also, the activity of IDH1/2 confers protection from oxidative damage, since NADPH is involved in reducing glutathione by glutathione reductase and α-KG is implicated as a potent antioxidant (7).

Mutations to IDH1/2 are important events in several types of cancers, including acute myeloid leukemia (AML), glioma, angio-immunoblastic T-cell lymphoma, chondrosarcoma, intrahepatic cholangiocarcinoma, and so on (8–11). In AML, IDH1/2 mutations were found in 16~33% patients, with R132H accounting for over 93% of IDH1 variants and R140Q/R172K being predominant in IDH2 variants (12, 13). These mutations gain a neomorphic catalytic function that converts α-KG to the oncometabolite R-2-hydroxyglutarate (2-HG) (14). The accumulating 2-HG, acting as a competitive inhibitor of α-KG, occupies the catalytic sites of multiple α-KG-dependent dioxygenases to inhibit their catalytic activity competitively. The potential targets of 2-HG inhibition that have acquired significant attention mainly include JmjC domain-containing histone demethylases (JmjC KDMs), tet methylcytosine dioxygenase 2 (TET2), and prolyl hydroxylases (PHD). JmjC KDMs and TET2 regulate gene expression by participating in histone and DNA demethylation, respectively, which contributes to the blockade of cell differentiation and the development of cancers. In addition, PHDs, the regulatory proteins catalyzing degradation of hypoxia-inducible factor 1α (HIF-1α), also can be inhibited by 2-HG that promotes oncogenesis and tumor progression (15).

Multiple studies have investigated the impact of IDH1/2 mutations on the prognosis of AML, with inconclusive results. Generally, IDH1 mutations are associated with an inferior outcome and IDH2 mutations are associated with a relatively favorable prognosis in AML (12, 16–19). In AML, mutant IDH1/2 serves as a poor prognostic factor in cytogenetically normal (CN)-AML with mutant NPM1 without FLT3-ITD (12). Several studies analyzed the specific clinical characteristics of AML patients with IDH1/2 mutations, and reported that IDH1/2 mutations are associated with old age, low WBC, high platelets, normal cytogenetics, and mutant NPM1 (12).

The discovery of IDH mutations in cancers promotes the rapid development of targeted inhibitors. Enasidenib (AG-221) and ivosidenib (AG-120) are inhibitors approved by FDA for the treatment of refractory or relapsed R/R AML with IDH2 or IDH1 mutations (20). Vorasidenib is a potent, oral, brain-penetrant dual inhibitor targeting both IDH1 and IDH2 mutants, which is undergoing a phase III INDIGO study (NCT04164901) in patients with residual or recurrent grade II glioma (21). A first-in-human phase I study (NCT02492737) of vorasidenib demonstrated well tolerability and preliminary antitumor activity in patients with low-grade gliomas (22).

Enasidenib and ivosidenib are first-in-class small molecule inhibitors targeting mutant IDH2 or IDH1. Biochemical and cellular analyses showed that ivosidenib was a highly selective inhibitor of IDH1, with no inhibition to IDH2 at micromolar concentrations. Preclinical data demonstrated that the treatment of ivosidenib significantly decreases the level of 2-HG in tumor models and promotes differentiation of primary human AML blast cells (23). Notably, ivosidenib and enasidenib demonstrated excellent clinical efficacy in IDH1/2-mutated R/R AML patients, with overall response rates (ORRs) of 41.6% and 40.3%, respectively, total CR rates of 21.6% and 19.3%, respectively, and median overall survival (mOS) of 8.8 months and 9.3 months, respectively. The median event-free survival (mEFS) duration for enasidenib-treated AML patients was 6.4 months. Vorasidenib showed preliminary antitumor activity in recurrent or progressive non-enhancing IDH1/2-mutated low-grade glioma with an objective response rate of 18% and a median progression-free survival of 36.8 months.

**Figure 1:**
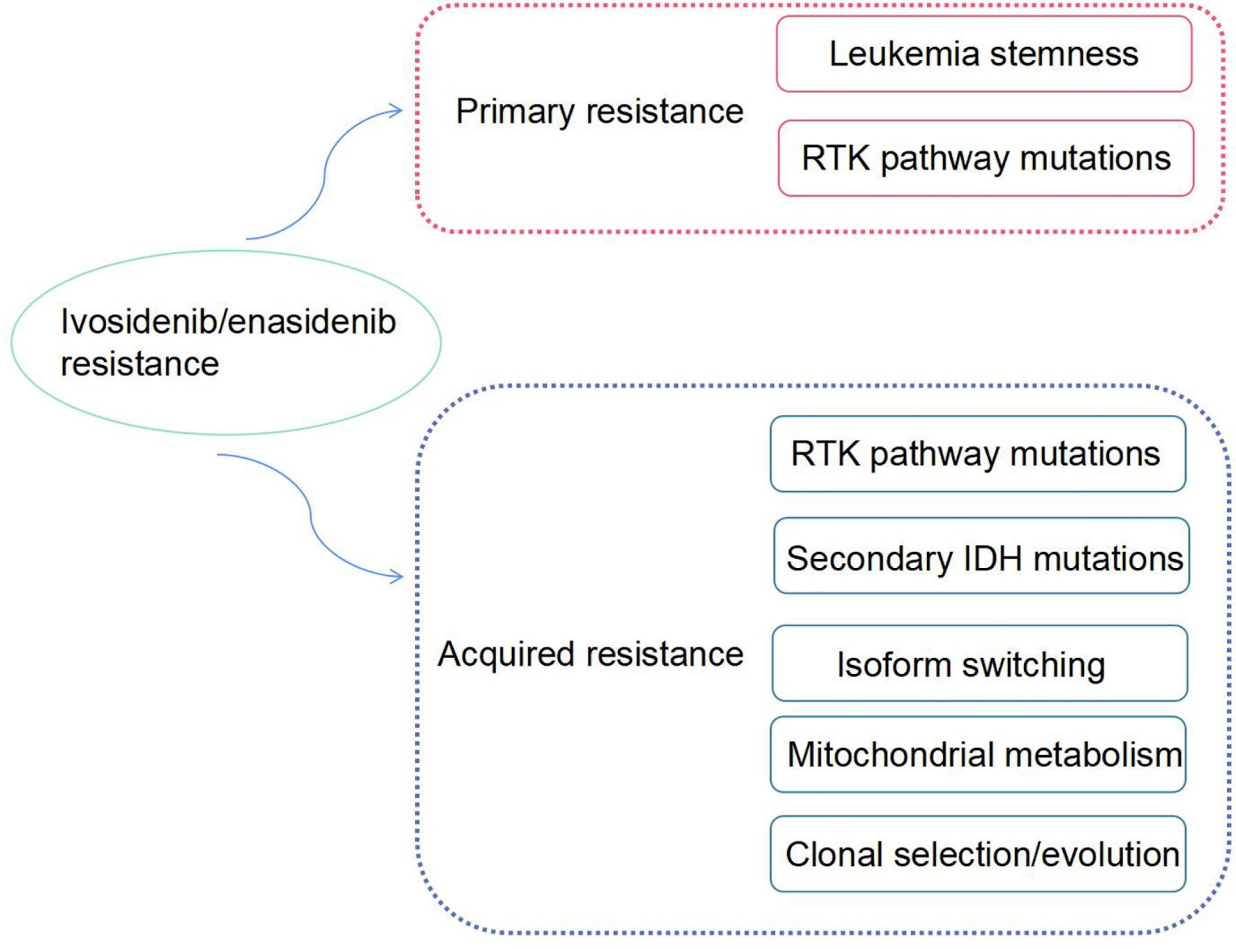
The mechanisms of ivosidenib/enasidenib resistance in AML.

Although enasidenib or ivosidenib have remarkable advantages as therapeutic agents in the treatment of R/R AML, drug-resistance inevitably occurs, rendering disease progression. Some AML patients with IDH mutations have no response to monotherapy with mIDH inhibitors, and some patients relapsed with elevated circulating levels of 2-HG and acquired resistance to IDH-targeted therapies. The mechanism of drug-resistance is complex. In this review, we discuss the mechanisms of resistance to enasidenib or ivosidenib and potential strategies to overcome these mechanisms (**Figure 1**).

## Secondary IDH Mutations

Allosteric inhibitors of IDH mutations exhibit similar binding characteristics, which was validated by using structure modeling. enasidenib was confirmed to bind to an allosteric site of mIDH2 within the homodimer interface, which stabilized the open conformation of mutant enzyme and suppressed production of 2-HG. Hydrogen bonds and hydrophobic interactions anchor enasidenib binding with Q316 residues of mIDH2 (24). In addition, interactions between enasidenib and other surrounding residuals including D312, W164, V294, V297, L298, V315, I319, and L320 also contribute to high inhibitory of potency (24). A recent case report showed two patients with gain resistance to enasidenib relapsed for secondary IDH2 mutations including Q316E and I319M. The structure modeling revealed that Q316 mutation led to diminished hydrogen bonds between enasidenib and IDH2, whereas I319M mutation led to steric hindrance for the bulky side chain. Another study described an AML patient with initial IDH1-R132H mutation who had a clinical response to ivosidenib, followed by relapse. Subsequent sequencing showed emergence of a secondary IDH1-S280F known as the cause of drug-resistance. The structure modeling confirmed that S280F mutation created steric hindrance between ivosidenib and mIDH1 dimer interface. These studies provide direct evidence for the resistance mechanism of second-site mutations, such as Q316E and I319M in IDH2 mutations and S280F in IDH1 mutations, that result in therapeutic resistance.

## Leukemia Stemness

Accumulating evidence has confirmed that cancer derives from cancer stem cells, a population of self-renewal cells that contribute to resistance to multiple therapies (25). Multipronged genomic analysis reveals the promoters of genes related to transcriptional regulation of leukemia stemness exhibit significant hypermethylation, which is closely related to primary resistance to IDH inhibitors (26). The molecular drivers of hypermethylated phenotype including FOXC1, CD99 and DNMT3A are identified as critical regulators of leukemia stemness. Additionally, targeting sequencing indicated co-occurring mutations of transcription factors related to hematopoietic differentiation including RUNX1, CEBPA and GATA2, are also associated with significantly worse response to IDH inhibitors (IDHi). Multi-logistic regression analysis showed that increased stemness is one of the mechanisms of IDHi primary resistance, and the score of stemness can be used as a potential predictive biomarker for IDH inhibitor response (26).

The Wnt/β-catenin signaling pathway, key components of the cascade for maintaining cancer cell stemness, participates in diverse physiological processes including proliferation, differentiation, apoptosis, invasion, migration, and tissue homeostasis, and its deregulation is closely related to initiation and progression of tumors (27–29). Whereas, 2-HG induces the hypermethylated inhibitory signals of Wnt, which lead to the improvement of stemness (30). Accumulating 2-HG resulted from IDH mutations improves leukemia stemness to block cell differentiation, which induces primary resistance to IDH inhibitors.

## Isoform Switching

Both IDH1 and IDH2 mutations have been reported in cancers, whereas usually only one mutation is identified in a certain cancer (31, 32). An AML patient with IDH mutation is usually treated by one type of small molecular inhibitor, either enasidenib or ivosidenib, to block disease progression. However, Harding et al. described four patients identifying IDH mutation isoform switching, either from mutant IDH1 to mutant IDH2 or vice versa (33).

Importantly, in this case report, two R/R AML patients with initial IDH1-R132C mutation achieved durable remissions with therapy of ivosidenib, but leukemia cells recurred with emergence of neomorphic mutation IDH2-R140Q. The third patient who suffered from treatment-refractory intrahepatic cholangiocarcinoma with IDH1-R132C obtained a sustained partial response to ivosidenib. The disease progressed subsequently with emergence of a new IDH2-R172V mutation. The fourth case, an R/R AML patient with initial IDH2-R140Q mutation achieved a durable remission with enasidenib, but disease progression occured with emergence of a new IDH1-R132C mutation which was sensitive to combined blockade IDH1/2 by vorasidenib. The isoform switching from IDH1 mutations to IDH2 mutations or vice versa were accompanied with elevated levels of 2-HG and disease progression. Therefore, isoform switching of IDH mutations is identified as one of the mechanisms of acquired resistance to IDH-targeted inhibitors. However, the precise frequency of isoform switching of IDH mutation remain unclear, which is essential to be determined in studies with large populations.

## RTK Pathway Mutations

Choe et al. performed a comprehensive genomic analysis in a large population of R/R AML patients carrying IDH mutations who were treated by ivosidenib, and confirmed that RTK pathway mutations are associated with primary resistance to ivosidenib. They performed co-occurring mutation profiling in these patients by NGS and found that baseline mutations of the individual RTK pathway genes including NRAS and PTPN11, and in the grouped RTK pathway genes including NRAS, KRAS, PTPN11, KIT, and FLT3, were implicated with an importantly lower likelihood of achievement of CR or CRh as a best response to ivosidenib (34). These results are consistent with previous work, in which NRAS mutation is associated with a worse response to enasidenib in R/R AML patients with mIDH2 (35). Therefore, co-occurring RTK pathway mutation is one of the mechanisms of primary resistance to mIDH inhibitors.

Additionally, Choe et al. observed emergence of RTK pathway mutations in about 35% relapsed cases achieving CR or CRh after monotherapy of ivosidenib, suggesting RTK pathway mutations are also associated with acquired resistance to ivosidenib (34). The biological processes that explain why RTK pathway mutations are implicated in both primary and acquired resistance to mIDH inhibitors are unclear. Several hypotheses were raised. First, it may be that the proliferative and prosurvival effects of RTK pathway activation are sufficiently strong oncogenic signals to reduce dependency on 2-HG. Second, it is possible that RTK pathway-activating mutations contribute to a differentiation block that remains enforced after initiation of ivosidenib treatment. A third hypothesis is that IDH1/2 mutations result in activation of some components of RTK signaling, which would not be reversed by ivosidenib in cases with co-occurring RTK pathway mutations.

## Mitochondrial Metabolism

Metabolic adaptations derived from changes of energy and intermediary metabolism in cancer cells are thought to meet biosynthetic and energetic requirements for proliferation (36). IHD1/2 play crucial roles in cell metabolism including Krebs cycle, cytosolic and mitochondrial redox, (oxidative phosphorylation) OxPHOS, and anabolism such as lipid biosynthesis. A better understanding of contributions of IDH mutations to metabolism and metabolic homeostasis may promote promising therapeutic strategies. Recently, several studies demonstrated that cancer cells carrying IDH mutations display some metabolic specificities, especially enhanced mitochondrial oxidative metabolism compared with wild-type cancer cells, and these cells tend to show vulnerability to mitochondrial inhibition (37–43).

Stuani et al. found AML patients with IDH mutations exhibited an enhanced mitochondrial oxidative metabolism which supports resistance to IDH mutation inhibitors. They performed multi-omics and functional approaches to investigate the mechanism of resistance. While IDH1 mutant inhibitor reduced 2-HG oncometabolite and CEBPα methylation, it failed to reverse (fatty acid β-oxidation) FAO and OxPHOS. OxPHOS, as a master regulator of mitochondrial biogenesis, and biosynthesis or degradation of FA, activation of PGC1α (peroxisome proliferator-activated receptor-γ coactivator-1) was not reversed after the inhibition of mIDH1. Importantly, FAO is a crucial biochemical process for sustaining OxPHOS and mitochondrial function in AML cells. Analysis of transcriptomic data from four clinical trials in 10 resistant patients demonstrated that genes associated with high OxPHOS function are enriched. Collectively, the OxPHOS phenotype was also confirmed as a nongenetic mechanism of IDHi resistance.

## Clonal Selection/Evolution

Accumulating emergence of somatic mutations in cancers promote the development of clonal heterogeneity (44). AML has been firmly established as a highly dynamic oligoclonal disease by using single-cell DNA sequencing approaches (45, 46). A good understanding of evolution of clonal heterogeneity is helpful to make a precise investigation of mechanisms of drug resistance.

Quek et al. studied the clonal basis of response and acquired resistance to enasidenib treatment (47). An analysis of paired diagnosis/relapse samples did not identify second site mutations in IDH2 at relapse. Instead, relapse arose by clonal evolution, or selection, of terminal or ancestral clones, highlighting multiple bypass pathways that could potentially be targeted to restore differentiation arrest. The increased variant allele frequency (VAF) of colony stimulating factor 3 receptor (CSF3R), (FMS-like tyrosine kinase 3) FLT3, and Cbl proto-oncogene (CBL), were identified as potential risks for acquired resistance. Relapse is also associated with concurrent mutations in U2 small nuclear RNA auxiliary factor 1 (U2AF1) and hematopoietic transcription factors, including RUNX1, BCL6 corepressor like 1 (BCORL1), GATA2, and BAF chromatin remodeling complex subunit BCL11A (BCL11A). The deletion of all or part of chromosome 7 is a risk factor for relapse after enasidenib treatment. Less reported variations in other genes including nuclear factor kappa B subunit 1 (NFKB1), DEAD-box helicase 1 (DDX1), microtubule associated scaffold protein 1 (MTUS1), DEAH-box helicase 15 (DHX15), and DEAF1 transcription factor (DEAF1), contribute to clonal evolution related to relapse. Mutations in DHX15 (R222G) and DDX1 (G699A) are notable due to their vital role in altering RNA splicing.

## Conclusion

In summary, this review concludes the resistance mechanisms of approved mIDH1/2 inhibitors and the increased understanding of drug-resistance mechanism would promote the development of corresponding strategies. To better investigate resistance mechanisms, development of novel effective approaches for detecting gene mutations is extremely important, such as next generation sequencing (NGS) and single-cell RNA sequencing (scRNA-seq). Future strategies should pay attention to the development of rational combination therapies with mIDH inhibitors or agents which can overcome the resistance to improve the response duration. The emerging biological mechanisms and clinical insights into these issues will provide guidance for rational treatment in the future.

## Author Contributions

(I) Conception and design: YC, CC, and YP; (II) Administrative support: None; (III) Provision of study materials or patients: None; (IV) Collection and assembly of data: None; (V) Data analysis and interpretation: None; (VI) Manuscript writing: All authors; (VII) Final approval of manuscript: All authors.

## Funding

We thank 100 Top Talents Program of Sun Yat-sen University,National Natural Science Foundation of China (NSFC, Grant No. 82000150), and Sanming Project of Medicine in Shenzhen (No. SZSM201911004) for supporting the manuscript preparation and publication.

## Conflict of Interest

The authors declare that the research was conducted in the absence of any commercial or financial relationships that could be construed as a potential conflict of interest.

## Publisher’s Note

All claims expressed in this article are solely those of the authors and do not necessarily represent those of their affiliated organizations, or those of the publisher, the editors and the reviewers. Any product that may be evaluated in this article, or claim that may be made by its manufacturer, is not guaranteed or endorsed by the publisher.
